# A Potential Screening Index of Corneal Biomechanics in Healthy Subjects, Forme Fruste Keratoconus Patients and Clinical Keratoconus Patients

**DOI:** 10.3389/fbioe.2021.766605

**Published:** 2021-12-23

**Authors:** Lei Tian, Xiao Qin, Hui Zhang, Di Zhang, Li-Li Guo, Hai-Xia Zhang, Ying Wu, Ying Jie, Lin Li

**Affiliations:** ^1^ Beijing Institute of Ophthalmology, Beijing Tongren Eye Center, Beijing Tongren Hospital, Capital Medical University and Beijing Ophthalmology and Visual Sciences Key Laboratory, Beijing, China; ^2^ Beijing Advanced Innovation Center for Big Data-Based Precision Medicine, Beijing Tongren Hospital, Beihang University & Capital Medical University, Beijing, China; ^3^ Beijing Key Laboratory of Fundamental Research on Biomechanics in Clinical Application, Capital Medical University, Beijing, China; ^4^ School of Biomedical Engineering, Capital Medical University, Beijing, China; ^5^ Department of Otolaryngology, Peking Union Medical College Hospital, Beijing, China; ^6^ The First People’s Hospital of Xuzhou, Xuzhou, China; ^7^ Department of Ophthalmology, Chinese People’s Liberation Army General Hospital, Beijing, China

**Keywords:** forme fruste keratoconus, clinical keratoconus, corneal visualization Scheimpflug technology, corneal elastic modulus, dynamic corneal response parameters

## Abstract

**Purpose:** This study aims to evaluate the validity of corneal elastic modulus (E) calculated from corneal visualization Scheimpflug technology (Corvis ST) in diagnosing keratoconus (KC) and forme fruste keratoconus (FFKC).

**Methods:** Fifty KC patients (50 eyes), 36 FFKC patients (36 eyes, the eyes were without morphological abnormality, while the contralateral eye was diagnosed as clinical keratoconus), and 50 healthy patients (50 eyes) were enrolled and underwent Corvis measurements. We calculated E according to the relation between airpuff force and corneal apical displacement. One-way analysis of variance (ANOVA) and receiver operating characteristic (ROC) curve analysis were used to identify the predictive accuracy of the E and other dynamic corneal response (DCR) parameters. Besides, we used backpropagation (BP) neural network to establish the keratoconus diagnosis model.

**Results:** 1) There was significant difference between KC and healthy subjects in the following DCR parameters: the first/second applanation time (A1T/A2T), velocity at first/second applanation (A1V/A2V), the highest concavity time (HCT), peak distance (PD), deformation amplitude (DA), Ambrosio relational thickness to the horizontal profile (ARTh). 2) A1T and E were smaller in FFKC and KC compared with healthy subjects. 3) ROC analysis showed that E (AUC = 0.746) was more accurate than other DCR parameters in detecting FFKC (AUC of these DCR parameters was not more than 0.719). 4) Keratoconus diagnosis model by BP neural network showed a more accurate diagnostic efficiency of 92.5%. The ROC analysis showed that the predicted value (AUC = 0.877) of BP neural network model was more sensitive in the detection FFKC than the Corvis built-in parameters CBI (AUC = 0.610, *p* = 0.041) and TBI (AUC = 0.659, *p* = 0.034).

**Conclusion:** Corneal elastic modulus was found to have improved predictability in detecting FFKC patients from healthy subjects and may be used as an additional parameter for the diagnosis of keratoconus.

## Introduction

Keratoconus (KC) presents as a progressive, non-inflammatory disease with a strong genetic component in which the cornea thins locally and forms into a conical shape. KC results in irregular astigmatism, loss of visual acuity and corneal bulge (Krachmer et al., 1984). Although KC is a bilateral disease, it may be a very asymmetric ectasia with a complete absence of, or very subtle variations commonly referred to as, “unilateral keratoconus’’ (McMahon et al., 2006; Randleman et al., 2008). When observed for a sufficient follow-up time, the disease will likely occur in their fellow eyes, at which point the eyes are referred to as “forme fruste keratoconus” or “subclinical keratoconus” (Rabinowitz et al., 1993; Holland et al., 1997; Li et al., 2004; Klyce 2009; Fontes et al., 2010). The etiology of keratoconus is poorly understood.

The diagnosis of keratoconus is mainly based on the abnormal corneal topography in keratoconus patients at present. Pentacam was one of the most widely used device for detection of abnormal corneas, while it has been hypothesized that corneal biomechanical destabilization in keratoconus patients may emerge prior to the corneal abnormal topography (Elham et al., 2017). As corneal topographical variations might be detectable before the tomographic and clinical signs of KC, corneal biomechanical properties might also be able to detect forme fruste keratoconus (Meek et al., 2005; Dupps and Wilson 2006; Morishige et al., 2007; Sinha Roy et al., 2013; Vellara and Patel 2015). Therefore, an increasing number of ophthalmologists and researchers in the field expect to determine corneal biomechanical properties through clinical measurements alone.

As a transparent biological soft tissue with complex material properties, the cornea is a nonlinear elastic, viscoelastic and anisotropic material. At present, corneal biomechanical experiments *in vitro*, such as corneal strip uniaxial tensile tests (Elsheikh and Anderson, 2005; Hatami-Marbini and Rahimi 2015a; Hatami-Marbini and Rahimi 2015b), corneal swelling tests (Elsheikh et al., 2007; Elsheikh et al., 2008; Kling et al., 2010) and corneal indentation tests (Ahearne et al., 2007; Wang et al., 2016) have been used to measure corneal biomechanical properties directly. Corneal elastic modulus can be extracted from these experiments effectively and found to be a function of strain. However, these experiments cannot be carried out in clinical settings directly.

Two devices have been widely used in measuring corneal biomechanics *in vivo*, corneal visualization Scheimpflug technology (Corvis ST) and ocular response analyzer (ORA). Both devices evaluate corneal biomechanical properties based on corneal response under rapid airpuff. Parameters provided by these devices have shown their values in diagnosing preliminarily keratoconus (Ayar et al., 2015; Elham et al., 2017; Koc et al., 2019; Atalay et al., 2020). Although parameters of these two devices are not only related to corneal biomechanics, they are highly confounded by corneal geometrical parameters and intraocular pressure (IOP) (Vinciguerra et al., 2016; Wang et al., 2016; Wu et al., 2016; Nemeth et al., 2017; Herber et al., 2019). Compared with ORA, Corvis measurements provided corneal deformations under airpuff, which make them evaluate the corneal biomechanical properties more intuitively.

The Corvis will be better applied in the clinic if the corneal typical biomechanical parameters (such as corneal elastic), which were not influenced by corneal geometrical parameters or IOP can be obtained directly by Corvis test. More and more studies have placed great concerns on extracting corneal biomechanical parameters from Corvis parameters directly (Wang et al., 2016; Shih et al., 2017). In the latest version of the Corvis software, a new parameter, the Stiffness Parameter (SP-A1), has been provided to reflect corneal stiffness. Shih et al. proposed a method to determine corneal elastic modulus according to the energy conversion during Corvis measurement (Shih et al., 2017). These new parameters may provide more accurate biomechanical information for diagnosing keratoconus. While the early diagnosis of keratoconus remains challenging and represents a significant area of interest, the technique of corneal tomography remains the diagnostic mode of choice (Shen et al., 2019). The combination of corneal mechanical and topographic parameters is expected to improve the efficiency of the early diagnosis of keratoconus. At present, more and more researchers and ophthalmologists are committed to relevant research.

Studies have found that the cornea can be regarded as a linear elastic material under physiological state (Zhang et al., 2018), so the mechanical properties of cornea under physiological state may be described by elastic modulus, or Young’s modulus, according to most of the concerned researchers. In our previous study, we proposed an effective method to evaluate the corneal elastic modulus (E) based on Corvis ST and in accordance with Reissner’s theory (Reissner, 1946). tThe calculated E was found less influenced by IOP and corneal geometrical parameters (Qin et al., 2019). In this current study, the sensitivity of the calculated corneal elastic modulus to distinguish forme fruste keratoconus patients and clinical keratoconus patients from healthy subjects are reported.

## Methods

### Subjects and measurements

Fifty clinical keratoconus patients (50 eyes), 36 forme fruste keratoconus patients (36 eyes), and 50 healthy subjects (50 eyes) were enrolled in the study. Subjects who had ocular diseases other than keratoconus, had corneal or ocular surgery history, or had systemic diseases that may affect the ocular function were excluded from this study. All participants had removed their soft contact lenses or rigid contact lenses at least 1 month before examination. A comprehensive ophthalmologic examination and a standardized interview were performed for all participants. All ophthalmologic examinations were performed at the same time to exclude the possible influence of diurnal fluctuation. The ophthalmic examinations included visual quality examination, slit-lamp microscopy examination, and fundus examination. All of keratoconus diagnosis was made by experienced ophthalmologists from Beijing Tongren Hospital.

For all subjects, Pentacam (Oculus Optikgeräte GmbH, Wetzlar, Germany) measurement was carried out to obtain corneal topography and tomography. Corvis ST measurements were carried out to obtain corneal biomechanical parameters. The Pentacam reconstructs a three-dimensional image of the entire anterior segment of the eye from the anterior surface of the cornea to the posterior surface of the lens by utilizing the high-speed rotating Scheimpflug system. Details and principles of the Pentacam are described by Fontes et al. (2010). Only scans that the Pentacam “quality specification” (QS) function determined as “OK” were included for analysis. According to our previous studies, B. Ele.Th, K_max_, Pachy_min_ et al. were sensitive to keratoconus (Zhang et al., 2021) and used as corneal topographic parameters in this study. The Corvis evaluates the dynamic corneal deformation response to an airpuff. Details and principles of the Corvis ST are described by Catalan-Lopez et al. (2018). In Corvis, the Corvis biomechanical index (CBI) and the tomographic and biomechanical index (TBI) were two comprehensive parameters reported to be sensitive to keratoconus. Only measurements where the “quality specification” read OK were accepted. If the comment was marked as yellow or red, the examination was repeated. The meaning of the partial parameters provided by Corvis and Pentacam are in Table 1. The bIOP reading from Corvis was used for the relevant data analysis.

**TABLE 1:** Abbreviations of Corvis and Pentacam output parameters.

Parameters short name	Description
IOP	Intraocular pressure (mmHg)
BIOP	Biomechanically corrected intraocular pressure
A1T	First applanation time (ms)
A1V	Velocity at first applanation (m/s)
A2T	Second applanation time (ms)
A2V	Velocity at second applanation (m/s)
HCT	Highest concavity time (ms)
DA	The maximum deformation amplitude (mm)
DARatio1	Ratio between deformation amplitude at apex and at 1 mm nasal and temporal
DARatio2	Ratio between deformation amplitude at apex and at 2 mm nasal and temporal
PD	Peak distance (mm)
SPA1	Adjusted pressure at (A1-bIOP)/A1 deflection amplitude
ARTh	Ambrosio relational thickness to the horizontal profile
TBI	The tomographic and biomechanical index
CBI	The corvis biomechanical index
B.Ele.Th	The elevation of the back surface at the thinnest location
Kmax	Maximum keratometry from the anterior corneal surface
Pachymin	Pachymetry at the thinnest point

The clinical keratoconus was diagnosed as follows: a) There was a scissoring reflex on retinoscopic or red reflex on ophthalmoscopic; b) The corneal topography results showed central or paracentral steepening. c) One or more of the following was found in slit-lamp examination: Vogt’s striae, Fleischer’s ring with an arc >2 mm, and corneal scarring consistent with keratoconus. In patients wherein only one eye was diagnosed as keratoconus, the keratoconus eye was included in the clinical keratoconus group, and the other eye was included in the forme fruste keratoconus (FFKC) group. There was no abnormal or suspect tomography characteristic in FFKC. For participants who were diagnosed with keratoconus in both eyes, one eye was selected randomly included in the clinical keratoconus group. For healthy subjects, one eye was selected randomly and included in the healthy group.

Data were collected from August to November 2019 at the Beijing Tongren Hospital, Beijing, China. All subjects were informed about the consent and had signed the informed consent form before the examination. The informed consent form was in compliance with the tenets of the Declaration of Helsinki. This study was approved by the institutional review board of the Beijing Tongren Hospital, Beijing Institute of Ophthalmology, Beijing, China.

### Determination of corneal elastic modulus

The method to evaluate the corneal elastic modulus has been proposed in our previous study (Qin et al., 2019). Corvis measurements were regarded as indentation experiments, in which the rapid airpuff was taken as a surface pressure acted on the corneal apex, and corneal deformation was recorded by the Scheimpflug camera. The airpuff amplitude with time was reported by Wang et al.,(2016), and the first applanation radius was regarded as the radius of the airpuff (*r*_*p*_). The corneal apical displacements were detected based on the edge detection of corneal anterior surface, which excluded the movement of the whole eyeball. When we take the cornea as a shallow spherical shell, we can determine the corneal elastic modulus according to the relation between the airpuff forces and corneal apical displacement presented in Eq. 1.

$$E=\frac{\Delta f}{\Delta \delta }\frac{\left(R-t/2\right)\sqrt{12\left(1-\nu^{2}\right)}}{\pi \;t}\frac{1-c_{1}}{\mu^{2}}$$ (1)

$$\mu =r_{p}{\left[\frac{12\left(1-\upsilon^{2}\right)}{{\left(R-\frac{t}{2}\right)}^{2}t^{2}}\right]}^{1/4}$$ (2)

In Eq. 1 above, *f* is the airpuff force acted on the corneal apex, δ is the corneal apical displacement, *R* is the central corneal curvature radius, t  is the corneal central thickness, and *R* and *t* were extracted and calculated from the corneal anterior surface edge. As the airpuff force–corneal apical displacement curve was approximately linear when the corneal apical displacement was between 0.2 and 0.4 mm during loading process (Wang et al., 2016), Δf/Δδ (mN/mm) is the slope of this curve when displacement is between 0.2 and 0.4 mm, ν is the corneal Poisson’s ratio and was set to 0.49 in this study, μ is defined by Eq. 2, and $c_{1}$ is determined by ν,R,t, and the radius of the airpuff (*r*_*p*_) according to the equations reported in our previous study (Qin et al., 2019).

### Backpropagation neural network Establishment

Based on the one-way ANOVA results, we combined some sensitive topometric and tomographic parameters in Pentacam of keratoconus (B.Ele.Th, K_max_, Pachy_min_ et al.) and DCR parameters to establish the keratoconus diagnosis model with BP (backpropagation) neural network. The implementation software is Matlab 2018a (MathWorks, United States). Of the data, 70% was used as the training set, and 30% of the data was used as the verification set. Several neural network models were tested with different hidden layer numbers and hidden layer neurons, and the one with high accuracy was selected. After testing, a three-layer neural network was selected. The number of neurons in the input layer depends on the number of parameters with statistical differences among the three groups, and the number of neurons in the output layer is set as 1; trainlm is selected as the activation function because it is suitable for medium-sized networks and have the fastest convergence speed. The learning rate is set to 0.01, the target error is set to 0.005, and the maximum number of iterations is set to 1,000 times.

### Statistical analyses

Statistical software SPSS (SPSS Inc., Chicago, IL, USA) version 21.0 was used for the statistical analysis in this study. The Kolmogorov–Smirnov (K-S) test was used to examine the normal distribution of quantitative data, and the results were presented as the mean and standard deviation (SD). Paired *t*-test and Bland–Altman plots were used to compare the agreement of CCT and R provided by Pentacam and Corvis ST. Besides, intraclass correlation coefficient (ICC) and concordance correlation coefficient (CCC) were calculated to more deeply analyze the agreement. One-way analysis of variance (ANOVA) and the least significant difference (LSD) test was used to evaluate the differences among different groups. Receiver operating characteristic (ROC) curve analysis was used with the aim of identifying the predictive accuracy of the calculated corneal elastic modulus and other DCR parameters provided by Corvis to analyze the sensitivity and specificity of these parameters. CBI and TBI were reported as keratoconus-sensitive parameters provided by Corvis. The ROC curve analysis was also applied to compare the predicted value and TBI, CBI in screening FFKC patients. Besides, the ROC curve comparison was used to analyze the value of E in the further identification of keratoconus. It was considered statistically significant when the alpha value of *p* was less than 0.05.

## Results

**FIGURE 1:**
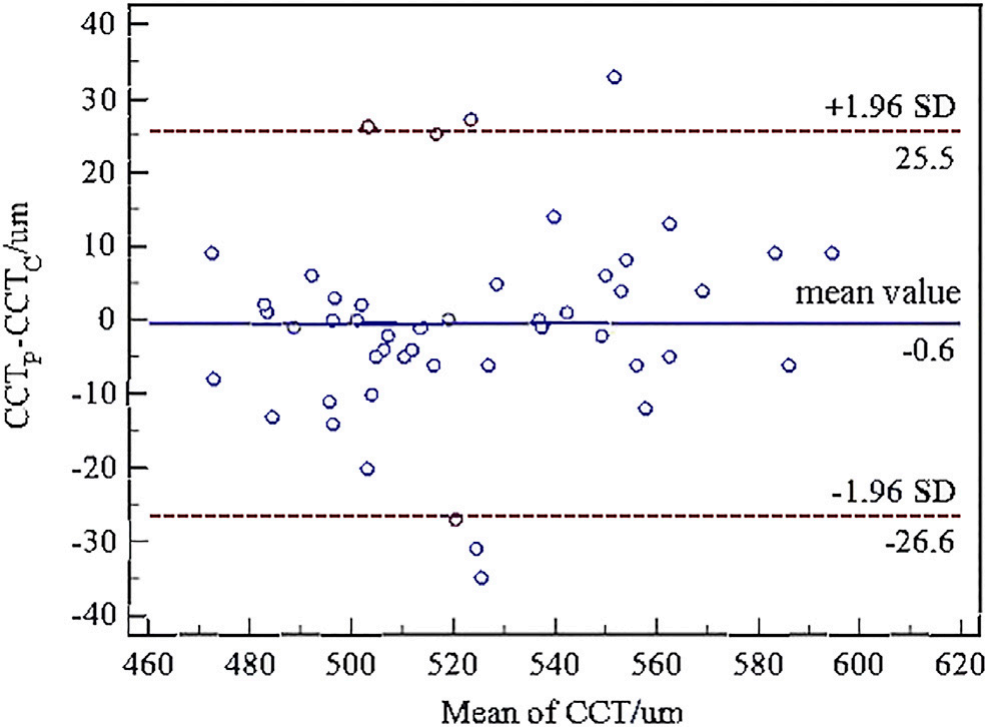
Bland–Altman plots of central corneal thickness (CCT) from Corvis ST and Pentacam. The *x*-axis was the mean value of the CCT from Corvis ST and Pentacam, and the *y*-axis was the difference of the CCT from Corvis ST and Pentacam. The horizontal dotted lines in the figure represent the 95% limits of agreement. The horizontal solid line represents the average value of the difference. Most of the differences are in this interval, which can be considered that the CCT from Corvis ST and Pentacam have good consistency.

**FIGURE 2:**
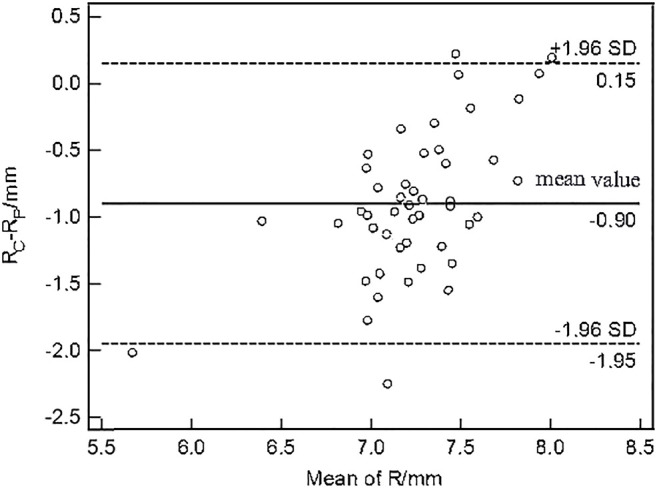
Bland–Altman plots of corneal curvature radius (R) from Corvis ST and Pentacam. The *x*-axis was the mean value of the R from Corvis ST and Pentacam, and the *y*-axis was the difference of the R from Corvis ST and Pentacam. The horizontal dotted lines in the figure represent the 95% limits of agreement. The horizontal solid line represents the average value of the difference. Most of the differences are in this interval, which can be considered that the R from Corvis ST and Pentacam have good consistency.

The paired *t*-test showed there was no significant difference between the central corneal thickness (CCT) provided Corvis and Pentacam (*p* = 0.293) in healthy subjects. Corneal curvature radius (R) provided by Corvis was about 0.9 mm smaller than that provided by Pentacam (*p* < 0.001). Figures 1, 2 showed the Bland–Altman plots of CCT and R provided by Pentacam and Corvis ST. The ICC of CCT and R between Corvis and Pentacam was 0.909 and 0.785, respectively, and the CCC was 0.909 and 0.832, respectively. These results remind us that the CCT and R calculated from Corvis ST were reliable. The mean value, SD of age range, bIOP, CCT, and R, obtained from Corvis measurements are shown in Table 2. The correlation between corneal elastic modulus (E) and bIOP, CCT, and R showed that E was significantly positively correlated with IOP in all of the three groups (*r* = 0.310, 0.384, 0.561 for healthy, KC, and FFKC groups, respectively; *p* < 0.05). E was positively correlated with R and negatively correlated with CCT, while the correlation was not statically significant (*p* > 0.05). These correlations were in agreement with the results of healthy subjects reported in our previous literature roughly (Qin et al., 2019). One-way ANOVA showed a significant difference in CCT and R among different groups. LSD tests results showed that there was no significant difference between the healthy and forme fruste keratoconus group for these parameters (*p* = 0.215, 0.276).

**TABLE 2:** Comparison of ocular morphology in different groups.

Parameters	Healthy group	KC group	FFKC group	Difference between healthy and KC group	Difference between healthy and FFKC group	Difference between KC and FFKC group	*p*
Age/years	25.9 ± 5.2	23.3 ± 7.7	23.6 ± 8.7	0.079	0.152	0.858	0.166
bIOP/mmHg	15.3 ± 2.0	14.3 ± 2.5	14.6 ± 1.8	0.120	0.119	0.456	0.059
CCT/μm	534.5 ± 34.6	462.8 ± 51.8	522.8 ± 39.5	<0.001	0.215	<0.001	<0.001*
R/mm	7.75 ± 0.92	5.36 ± 1.01	6.94 ± 0.95	<0.001	0.276	<0.001	<0.001*

Note. BIOP, biomechanical corrected IOP; R, corneal curvature radius; CCT, central corneal thickness; FFKC, forme fruste keratoconus; KC, keratoconus.

*There was statistical difference among different groups.

The DCR parameters that were significantly different among groups and the corneal elastic modulus are shown in Table 3. The LSD tests results showed that the A1T and E were smaller in the forme fruste keratoconus group than those in the healthy group (*p* < 0.001), while there was no significant difference seen between the healthy and forme fruste keratoconus group for the other DCR parameters (*p* > 0.05).

**TABLE 3:** Corneal biomechanical parameters in different groups.

Parameters	Healthy group	KC group	FFKC group	Difference between healthy and KC group	Difference between healthy and FFKC group	Difference between KC and FFKC group	*p*
A1T/ms	7.33 ± 0.23	6.82 ± 0.26	7.16 ± 0.20	<0.001	0.001	<0.001	<0.001
A1V/ m s^−1^	0.15 ± 0.02	0.19 ± 0.02	0.16 ± 0.02	<0.001	0.505	<0.001	<0.001
A2T/ms	21.84 ± 0.29	22.22 ± 0.97	21.90 ± 0.67	0.009	0.703	0.042	0.021
A2V/ m s^−1^	−0.28 ± 0.03	−0.35 ± 0.10	−0.28 ± 0.06	<0.001	0.653	<0.001	<0.001
HCT/ms	17.08 ± 0.40	16.85 ± 0.54	16.90 ± 0.44	0.016	0.078	0.651	0.042
PD/mm	5.21 ± 0.23	5.37 ± 0.23	5.25 ± 0.24	0.001	0.440	0.022	0.003
DA/mm	1.09 ± 0.08	1.35 ± 0.18	1.12 ± 0.09	<0.001	0.081	<0.001	<0.001
SP-A1	93.301 ± 14.487	41.581 ± 13.542	79.574 ± 14.127	<0.001	<0.001	<0.001	<0.001
ARTh	451.27 ± 112.74	193.17 ± 109.83	400.41 ± 97.93	<0.001	0.083	<0.001	<0.001
CBI	0.19 ± 0.27	0.97 ± 0.14	0.42 ± 0.41	<0.001	<0.001	<0.001	<0.001
STSC/mN mm^−1^	25.64 ± 1.79	15.88 ± 2.53	24.59 ± 3.85	<0.001	0.081	<0.001	<0.001
E/MPa	0.35 ± 0.04	0.16 ± 0.04	0.30 ± 0.08	<0.001	<0.001	<0.001	<0.001

**FIGURE 3:**
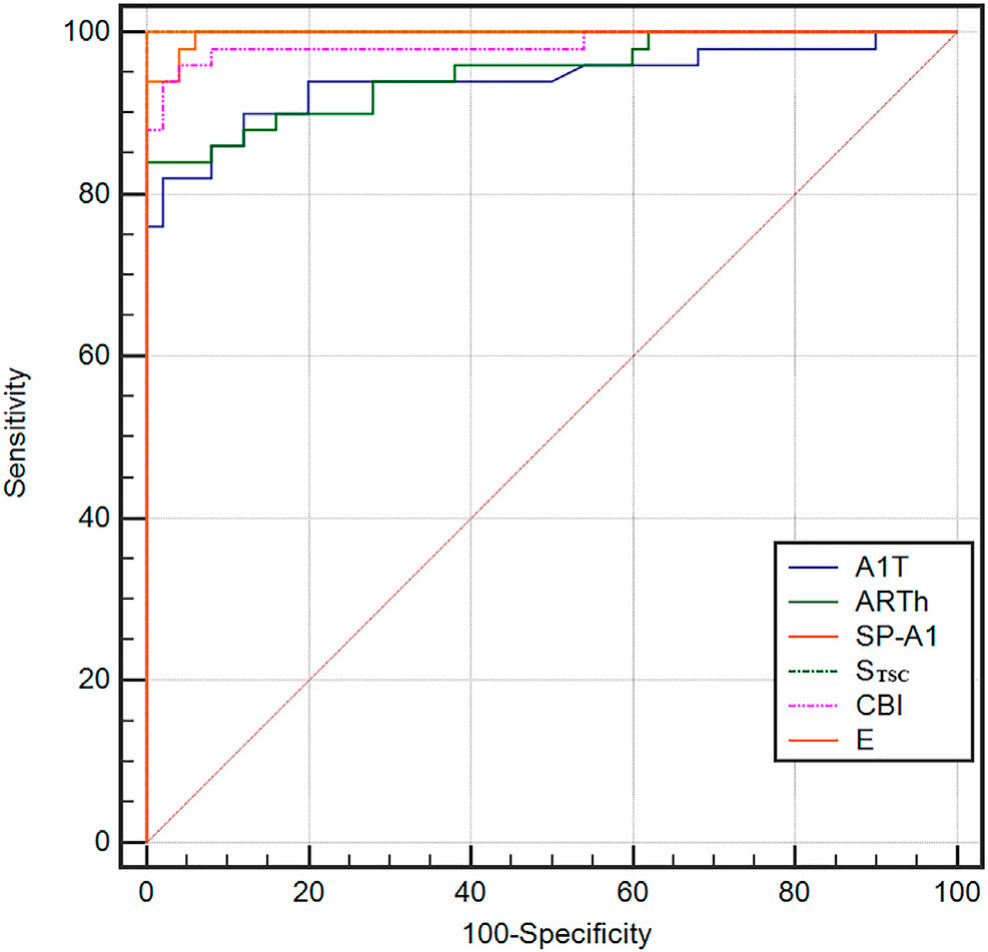
Receiver operating characteristic (ROC) curves for the dynamic corneal response (DCR) parameters provided by Corvis ST in the detection of clinical keratoconus. All of these parameters in the figure can diagnose clinical keratoconus with high specificity and sensitivity (AUC > 0.9).

**FIGURE 4:**
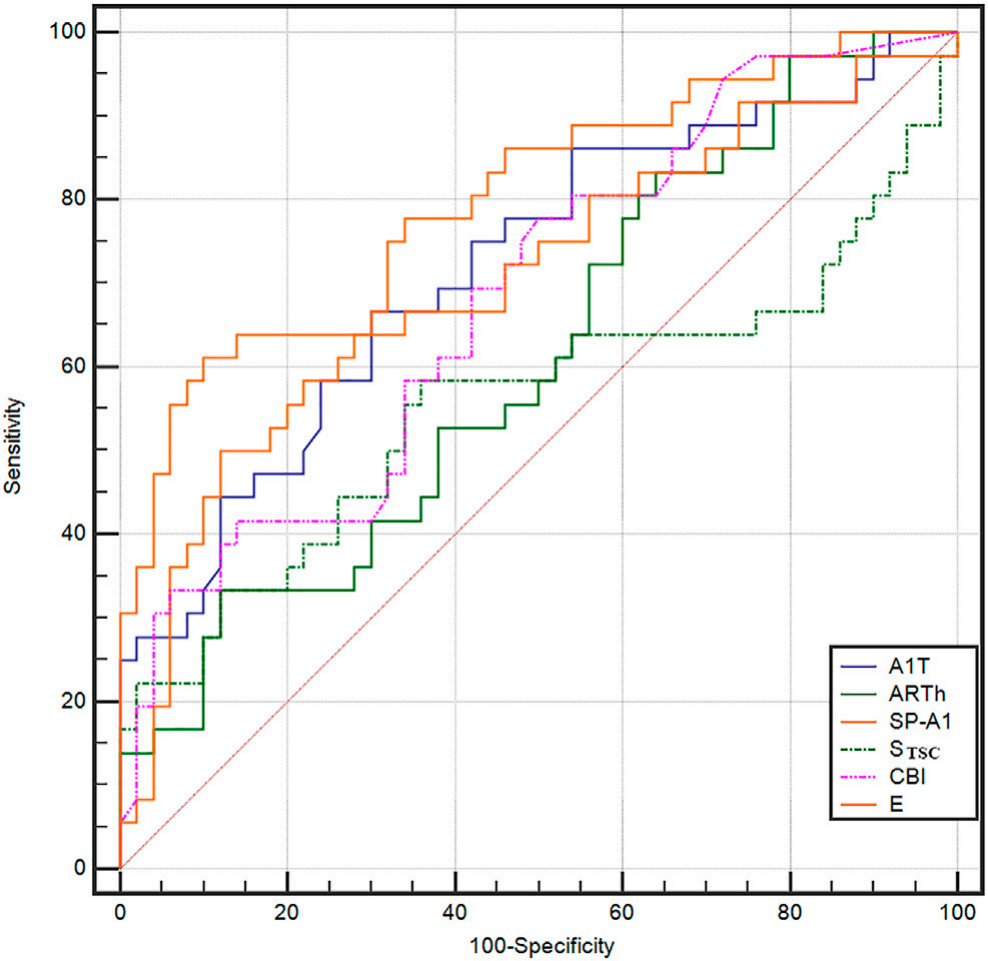
ROC curves for the DCR parameters provided by Corvis ST in the detection of forme fruste keratoconus. The AUC of E was 0.746, which was more accurate than the other parameters, such as the Stiffness Parameter (SP-A1; AUC = 0.710) and Corvis biomechanical index (CBI) (AUC = 0.684), in the diagnosis of forme fruste keratoconus (FFKC). The E can be a potential index in screening FFKC.

The ROC curve analysis was used to detect forme fruste keratoconus/clinic keratoconus from healthy subjects. The cutoff point, specificity, sensitivity, and AUC are shown in Table 4. Figures 3, 4 and show the ROC curves. Most of the parameters in Table 4 can diagnose clinical keratoconus with high specificity and sensitivity (AUC > 0.9), while E (AUC = 0.746) was more accurate than the other parameters, such as SP-A1 (AUC = 0.710, *p* = 0.084) and CBI (AUC = 0.684, *p* = 0.041) in the diagnosis of FFKC.

**TABLE 4:** Receiver operating characteristic (ROC) curve analysis results for the detection of KC and FFKC.

Parameters	FFKC				KC			
	Cutoff point	Specificity	Sensitivity	AUC	Cutoff point	Specificity	Sensitivity	AUC
A1T/ms	7.22	0.667	0.700	0.719	7.049	0.980	0.820	0.941
A1V/ m s^−1^	0.144	0.300	0.833	0.517	0.171	0.920	0.860	0.954
A2T/ms	22.236	0.960	0.278	0.599	22.128	0.900	0.780	0.856
A2V/ m s^−1^	0.303	0.880	0.417	0.599	0.317	0.940	0.900	0.926
PD/mm	5.517	0.920	0.222	0.543	5.32	0.720	0.580	0.678
DA/mm	1.195	0.980	0.278	0.603	1.179	0.960	0.920	0.984
SP-A1	88.191	0.620	0.778	0.710	57.526	1.000	0.940	0.997
ARTh	330.34	0.880	0.333	0.611	272.60	1.000	0.840	0.950
CBI	0.039	0.500	0.778	0.684	0.764	0.960	0.960	0.986
STSC/mN mm^−1^	25.27	0.583	0.640	0.556	22.21	1.000	1.000	1.000
E/MPa	0.31	0.649	0.860	0.746	0.245	1.000	1.000	1.000

**FIGURE 5:**
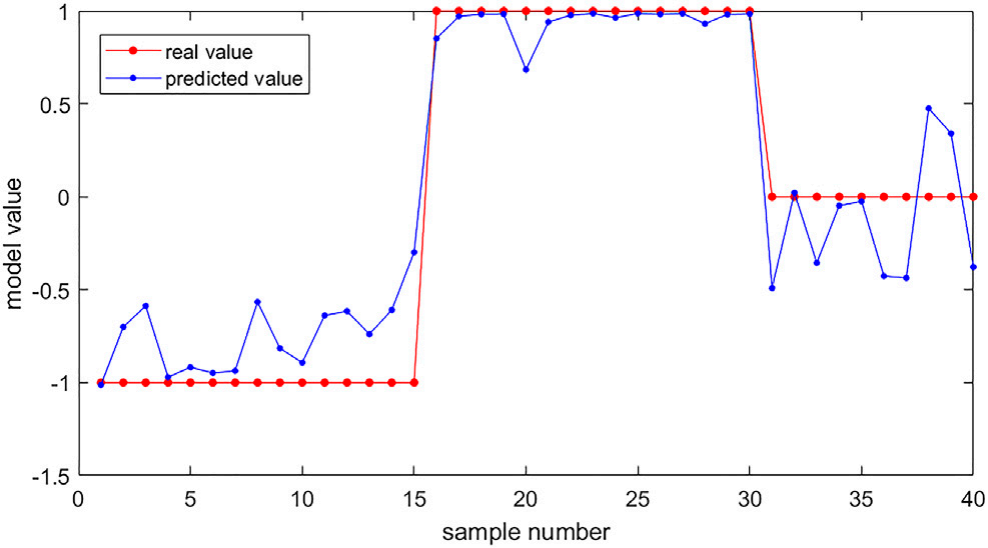
The results of keratoconus diagnosis with the keratoconus diagnosis model. Subjects (40) were used as the verification set. The red dots are the real value of the subjects according to their group (the healthy group was set to be 1, the FFKC group was set to be 0, and the KC group was set to be −1). The blue dots are the predicted value of the subjects calculated by the backpropagation (BP) neural network. We can predict the group of the subjects according to the predicted value (<−0.5: healthy group; −0.5∼0.5: FFKC group; >0.5: KC group).

**FIGURE 6:**
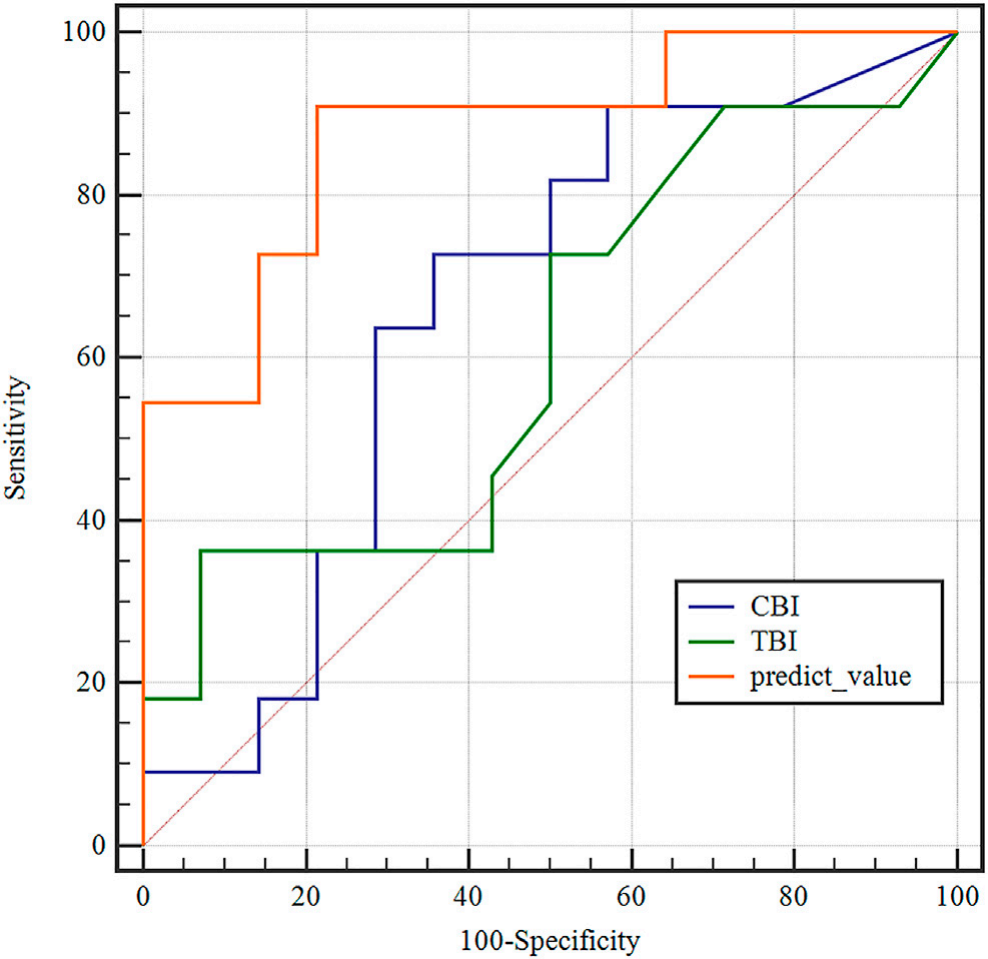
Receiver operating characteristic (ROC) curves of the predicted values of the BP neural network model and tomographic and biomechanical index (TBI), CBI in the detection of forme fruste keratoconus. The predicted value of the BP neural network model (AUC = 0.877) was more accurate than TBI (AUC = 0.659), CBI (AUC = 0.610) in the detection of FFKC.

According to one-way ANOVA, eight parameters provided by Corvis in Table 3 (A1T, A1V, A2T, A2V, SP-A1, PD, DA, ARTh) and three sensitive Pentacam parameters of keratoconus (B.Ele.Th, K_max_, Pachy_min_) and E were used to establish the keratoconus diagnosis model. According to the preliminary test, we selected a three-layer neural network, and the number of neuron nodes are 12, 5, and 1, respectively. Figure 5 and Table 5 show the results of the diagnosis. The diagnosis model has an accuracy of 92.5% in distinguishing healthy cornea, keratoconus in the frustration stage, and keratoconus in the clinical stage. Figure 6 shows the ROC curves of the predicted value provided by the keratoconus diagnosis model and TBI, CBI provided by Corvis in screening FFKC patients. Table 5 shows that our predicted value (AUC = 0.877) is more sensitive to FFKC than CBI (AUC = 0.610, *p* = 0.041) and TBI (AUC = 0.659, *p* = 0.034).

**TABLE 5:** ROC curve comparison for the detection of FFKC among the backpropagation (BP) neural network model model predicted value and TBI, CBI.

Parameters	Specificity	Sensitivity	AUC	ROC comparison with predicted value (*p*)
Predicted value of BP neural network model	0.800	0.909	0.877	—
CBI	0.500	0.771	0.610	0.041
TBI	0.428	0.829	0.659	0.034

## Discussion

Keratoconus (KC) is a progressive, non-inflammatory ectatic corneal disorder in which the normal cornea thins locally and forms into a conical shape. Abnormal corneal tomography is one of the most commonly used indexes for the diagnosis of keratoconus (Meek et al., 2005; Morishige et al., 2007). Histopathological studies have found the losses of the collagen fibrils and interfibrillary substance during the development of keratoconus. Besides, corneal collagen fibrils were found to slip in the stroma. These structural changes may result in the instability of corneal biomechanical properties. The instability of corneal biomechanics can consequently lead to changes in corneal tomography. Therefore, comprehending corneal biomechanics is important to describe and diagnose KC (Vinciguerra et al., 2017; Shao et al., 2019). In this study, the corneal elastic modulus was calculated from the Corvis measurements to characterize corneal biomechanical properties in patients with forme fruste keratoconus patients, clinical keratoconus patients, and healthy subjects. The results showed that corneas with keratoconus exhibited less elastic modulus than did IOP matched healthy control corneas. Besides, the results also showed that corneal biomechanical properties were altered in forme fruste keratoconus eyes. The results of the ROC curve analysis showed that the corneal elastic modulus has a good level of predictive accuracy in detecting forme fruste keratoconus from healthy corneas.

Several prior research studies have described corneal biomechanical properties in healthy and keratoconus subjects *in vivo* (Goebels et al., 2017; Hashemi et al., 2017; Herber et al., 2019). Hashemi et al. examined the diagnostic validity of the different corneal biomechanical parameters provided by ORA in detecting early keratoconus. This study demonstrated that the novel waveform-derived indices provided by ORA have important role in the detection of early keratoconus. Furthermore, the results showed that the p1area, p2area, h1, h2, dive1, mslew1, aspect1, aplhf, dslope1, and CRF had a high degree of sensitivity and specificity in detecting early keratoconus (Goebels et al., 2017; Hashemi et al., 2017), while the mechanical significance of these parameters derived from ORA was not clear, and there is a large cross in the range of these parameters between healthy subjects and FFKC patients. Elham et al. also assessed the capacity of Corvis in diagnosing keratoconus (Kozobolis et al., 2012; Elham et al., 2017). They found that some parameters, such as CCT, the highest concavity radius, and DA, were significantly different between healthy corneas and keratoconus. Studies on the corneal biomechanical properties of keratoconic eyes with different severity grades showed that both ORA and Corvis ST allowed for good differentiation between healthy eyes and keratoconic eyes with different severity grades (Koh et al., 2020).

Since forme fruste keratoconus eyes lacked any, or presented with very subtle changes in geometrical parameters, many built-in parameters of these devices cannot be utilized for diagnosing forme fruste keratoconus patients because of their strong correlation with corneal geometrical parameters (Shen et al., 2019). Thus, parameters directly describing the mechanical meaning could help in understanding the corneal biomechanical changes in early keratoconus patients better.

In this study, both the corneal elastic modulus and S_TSC_ were smaller in clinical keratoconus patients, which was an observation that agreed with our previous study (Wang et al., 2016). However, there was no significant difference between healthy subjects and forme fruste keratoconus patients for S_TSC_. In our last study, the corneal elastic modulus was calculated, and this value was found to be less correlated with IOP than S_TSC_ and corneal geometrical parameters (Qin et al., 2019).

According to the results of the ROC curve analysis, several parameters showed good predictability in distinguishing clinical keratoconus patients from healthy corneas (AUC > 0.9), which was agreed with the reported results (Kozobolis et al., 2012; Kozobolis et al., 2012; Elham et al., 2017; Elham et al., 2017). Corneal elastic modulus showed better predictability (AUC was 0.746) in distinguishing forme fruste keratoconus patients from healthy corneas. The LSD test and ROC curve analysis showed that the corneal elastic modulus was significantly smaller in the forme fruste keratoconus patients than in healthy patients. The results of the present study confirmed that the changes in corneal biomechanical properties arose in early keratoconus before the evident corneal geometry changes (Kozobolis et al., 2012). Several literature have reported the diagnostic validity of CBI in the detection of FFKC (Steinberg et al., 2018; Herber et al., 2019; Koh et al., 2020). In this study, the built-in parameters provided by Corvis ST Software were also compared between forme fruste keratoconus patients and healthy subjects, and the results showed that A1T was the best predictive parameter with an AUC of 0.719. A1T has been found to be an important parameter to reflect corneal biomechanical properties, while it was strongly correlated with IOP (Wang et al., 2017). In this study, there was no significant difference in IOP among the three groups, and the sensitivity and specificity of the A1T were similar to those of the corneal elastic modulus, which may demonstrate that A1T can reflect corneal elastic properties under normal IOP. The corneal elastic modulus showed better predictability in diagnosing keratoconus compared with the Corvis built-in parameters. The keratoconus diagnosis model combining E, DCR parameters, and corneal Pentacam parameters by the BP neural network showed a more accurate diagnostic efficiency of 92.5%. Besides, the ROC curve analysis results showed that the predicted value provided by BP neural network is more sensitive to FFKC than to TBI and CBI, while additional work based on more clinical data should be done in the future for clinical applications because of the limited amount of sample in this study.

One of the limitations was that the sample number in this study is small. More subjects may be necessary to verify the results of this study. Another limitation was that the parameters related to corneal viscoelasticity were not applied to detect forme fruste keratoconus patients from healthy subjects. Although the corneal elastic modulus showed better identifiability of forme fruste keratoconus, a great number of researchers have believed that corneal viscoelasticity is also important to diagnose KC. Thus, further study should be carried out to determine corneal viscoelastic parameters from Corvis measurements and to apply the outcome to detect KC by combining the results of this study in the future.

In conclusion, the corneal elastic modulus was calculated and compared in healthy subjects, forme fruste keratoconus patients, and clinical keratoconus patients. The corneal elastic modulus showed improved predictability in detecting forme fruste keratoconus patients compared with normal apparently healthy subjects, which might be used as an additional parameter for keratoconus diagnosis. Further study is needed to generate more accurate methods to diagnose forme fruste keratoconus patients when also combined with corneal biomechanical and corneal topography parameters.

## Data Availability

The raw data supporting the conclusion of this article will be made available by the authors, without undue reservation.
