# Corrigendum: A potential screening index of corneal biomechanics in healthy subjects, forme fruste keratoconus patients and clinical keratoconus patients

**DOI:** 10.3389/fbioe.2022.1011816

**Published:** 2022-08-30

**Authors:** Lei Tian, Xiao Qin, Hui Zhang, Di Zhang, Li-Li Guo, Hai-Xia Zhang, Ying Wu, Ying Jie, Lin Li

**Affiliations:** ^1^ Beijing Institute of Ophthalmology, Beijing Tongren Eye Center, Beijing Tongren Hospital, Capital Medical University and Beijing Ophthalmology and Visual Sciences Key Laboratory, Beijing, China; ^2^ Beijing Advanced Innovation Center for Big Data-Based Precision Medicine, Beijing Tongren Hospital, Beihang University and Capital Medical University, Beijing, China; ^3^ Beijing Key Laboratory of Fundamental Research on Biomechanics in Clinical Application, Capital Medical University, Beijing, China; ^4^ School of Biomedical Engineering, Capital Medical University, Beijing, China; ^5^ Department of Otolaryngology, Peking Union Medical College Hospital, Beijing, China; ^6^ The First People’s Hospital of Xuzhou, Xuzhou, China; ^7^ Department of Ophthalmology, Chinese People’s Liberation Army General Hospital, Beijing, China

**Keywords:** forme fruste keratoconus, clinical keratoconus, corneal visualization scheimpflug technology, corneal elastic modulus, dynamic corneal response parameters

In the published article, there was an error in affiliation 5. Instead of “Department of Ophthalmology, Chinese People’s Liberation Army General Hospital, Beijing, China”, it should be “Department of Otolaryngology, Peking Union Medical College Hospital, Beijing, China”.

The authors apologize for this error and state that this does not change the scientific conclusions of the article in any way. The original article has been updated.

